# P-1977. Six-Month Trajectory of Symptoms of COVID-19 Fatigue by Age and BNT162b2 COVID-19 Vaccination Status: A Prospective Study among Symptomatic US Adults Testing Positive for SARS-CoV-2 at a National Retail Pharmacy

**DOI:** 10.1093/ofid/ofae631.2135

**Published:** 2025-01-29

**Authors:** Manuela Di Fusco, Alexandra Berk, Kristen E Allen, Thomas M Porter, Mary B Alvarez, Joseph C Cappelleri, Mary M Moran, Xiaowu Sun

**Affiliations:** Pfizer Inc, New York, New York; CVS Health Clinical Trial Services, Woonsocket, Rhode Island; Pfizer, Inc., Minneapolis, Minnesota; Pfizer Inc., New York, New York; Pfizer Inc., New York, New York; Pfizer Inc., New York, New York; Pfizer Inc, New York, New York; CVS Health, Woonsocket, Rhode Island

## Abstract

**Background:**

Fatigue is one of the most common symptoms experienced by patients with COVID-19, potentially interfering with daily activities. This study assessed the 6-month trajectory of fatigue from time of testing across age and BNT162b2 BA.4/5 bivalent COVID-19 Vaccine strata.

**Figure 1.**
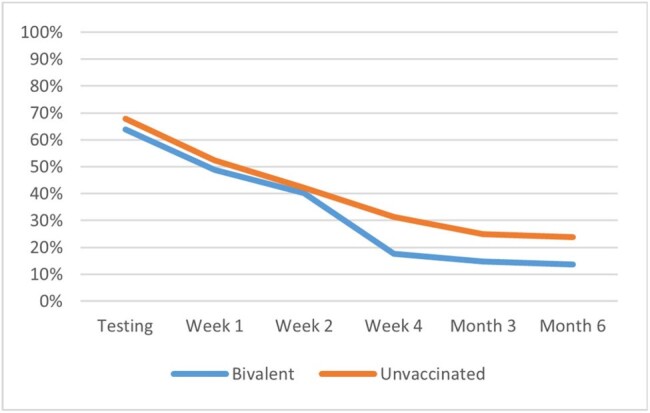
Prevalence of symptoms of fatigue across groups defined by age and BNT162b2 COVID-19 Vaccination status a) Overall cohort by BNT162b2 vaccination status

**Methods:**

Symptomatic US adults testing positive for SARS-CoV-2 at CVS Health were recruited between 03/02-05/18/2023 (CT.gov: NCT05160636). Study participants self-reported symptoms of fatigue via an online survey during the acute (testing day, Week 1, Week 2) and long-term phase (Week 4, Month 3 and 6 after testing). The prevalence of fatigue was evaluated at each time point in the overall cohort and across groups defined by vaccination status and age (older adults: ≥50; younger adults: < 50). Between-group differences were tested by using chi-square statistics.

**Figure:**
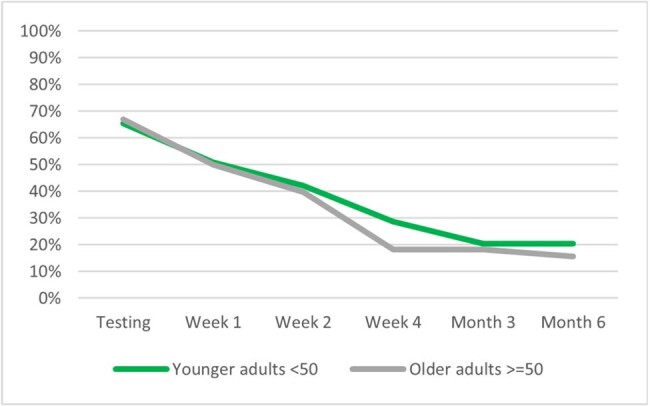

b) Overall cohort by age

**Results:**

The analyses included 643 study participants: 49% (316) vaccinated with BNT162b2 bivalent and 51% (327) unvaccinated; 43.1% (277) ≥50 and 56.9% (366) < 50 years old. A total of 424 (65.9%) participants self-reported symptoms of fatigue at time of testing. Their mean age was 46.5 years, 71.2% were female, 25.9% had ≥1 comorbidity, 43.2% had prior infection. The presence of fatigue was higher during the acute phase and remained prevalent during the long-term follow-up: 50.9% at Week 1, 41.1% at Week 2, 24.4% at Week 4, 19.6% at Month 3, and 18.5% at Month 6 (Figure 1a). While older and younger adults had similar prevalence of fatigue during the acute phase, at Week 4 the younger adults reported higher prevalence (28.7% vs 18.2%, p=0.007) (Figure 1b). In the overall cohort, and relatively consistently across age groups, those vaccinated with BNT162b2 reported significantly (p< 0.005) lower prevalence of fatigue at Week 4, Month 3 and 6 (Figures 1a, 1c, 1d).

**Figure:**
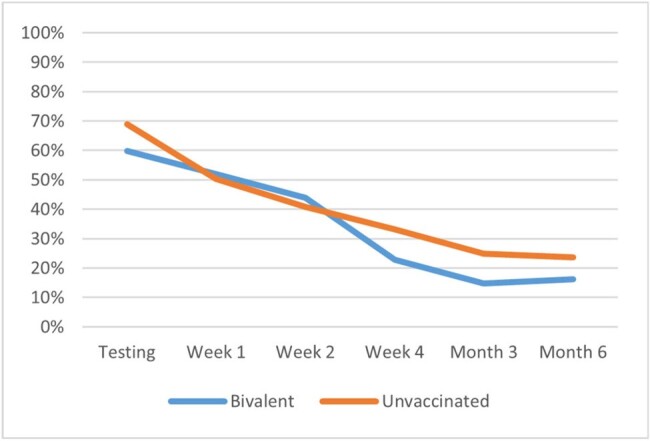

c) Younger adults (<50)

**Conclusion:**

This study found that fatigue is a highly prevalent symptom affecting two-thirds of symptomatic adults at time of testing. While prevalence declined over time, fatigue symptoms persisted beyond the acute phase, with 1 in 5 still affected at Month 6. Compared with those vaccinated with BNT162b2 bivalent, the prevalence of fatigue was higher among unvaccinated throughout the follow-up, reaffirming the value of being up-to-date with COVID-19 vaccination.

**Figure:**
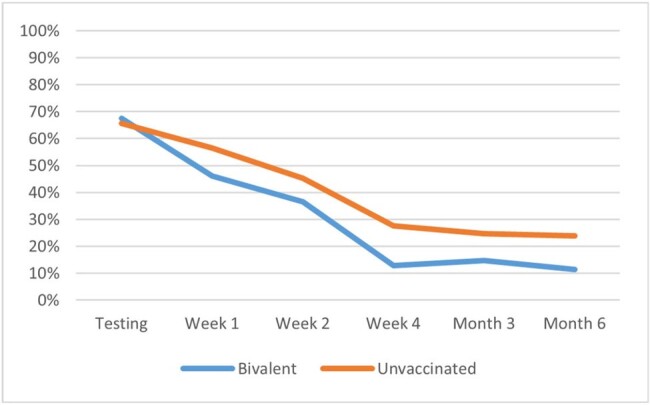

d) Older adults (≥50)

**Disclosures:**

Manuela Di Fusco, PhD, Pfizer Inc.: Employee|Pfizer Inc.: Stocks/Bonds (Public Company) Alexandra Berk, PhD, CVS Health: Employee|CVS Health: Stocks/Bonds (Public Company) Kristen E. Allen, MPH, Pfizer Inc.: Employee|Pfizer Inc.: Stocks/Bonds (Public Company) Thomas M. Porter, MPH, Pfizer Inc.: Employee|Pfizer Inc.: Stocks/Bonds (Public Company) Mary B. Alvarez, PharmD, Pfizer Inc.: Employee|Pfizer Inc.: Stocks/Bonds (Public Company) Joseph C. Cappelleri, PhD, Pfizer Inc.: Employee|Pfizer Inc.: Stocks/Bonds (Public Company) Mary M. Moran, MD, Pfizer Inc.: Employee|Pfizer Inc.: Stocks/Bonds (Public Company) Xiaowu Sun, PhD, CVS Health: Employee|CVS Health: Stocks/Bonds (Public Company)

